# Qualitative focus group study investigating experiences of accessing and engaging with social care services: perspectives of carers from diverse ethnic groups caring for stroke survivors

**DOI:** 10.1136/bmjopen-2015-009498

**Published:** 2016-01-29

**Authors:** Nan Greenwood, Jess Holley, Theresa Ellmers, Gill Mein, Geoffrey Cloud

**Affiliations:** 1 Faculty of Health, Social Care and Education, St George's University of London and Kingston University, London, UK; 2 Department of Neurology, St George's Healthcare NHS Trust, London, UK

**Keywords:** QUALITATIVE RESEARCH

## Abstract

**Objectives:**

Informal carers, often family members, play a vital role in supporting stroke survivors with post-stroke disability. As populations age, numbers of carers overall and those from minority ethnic groups in particular, are rising. Carers from all ethnic groups, but especially those from black and minority ethnic groups frequently fail to access support services, making understanding their experiences important. The study therefore explored the experiences of carers of stroke survivors aged 45+ years from 5 ethnic groups in accessing and receiving social care services after hospital discharge.

**Design:**

This qualitative study used 7 recorded focus groups with informal carers of stroke survivors. Data were analysed thematically focusing on similarities and differences between ethnic groups.

**Setting:**

Carers were recruited from voluntary sector organisations supporting carers, stroke survivors and black and minority ethnic groups in the UK.

**Participants:**

41 carers from 5 ethnic groups (Asian Indian, Asian Pakistani, black African, black Caribbean, white British) participated in the focus groups.

**Results:**

Several interconnected themes were identified including: the service gap between hospital discharge and home; carers as the best person to care and cultural aspects of caring and using services. Many themes were common to all the included ethnic groups but some related to specific groups.

**Conclusions:**

Across ethnic groups there were many similarities in the experiences of people caring for stroke survivors with complex, long-term care needs. Accessing services demands effort and persistence on carers’ part. If carers believe services are unsatisfactory or that they, rather than formal services, should be providing support for stroke survivors, they are unlikely to persist in their efforts. Cultural and language differences add to the challenges black and minority ethnic group carers face.

**Strengths and limitations of this study**

- We used a qualitative, in-depth approach with over 40 participants giving a detailed understanding of these carers’ experiences.
- We included minority ethnic and white ‘majority’ participants, which permitted direct comparison between these groups.
- However, all focus groups were conducted in English excluding carers with limited or no English. Finally, we only investigated five ethnic groups which are clearly very broad categories with inherent limitations.

## Introduction

Worldwide stroke is the major cause of long-term disability in adults^1^ and leaves many stroke survivors dependent on their families (who are often referred to as carers or caregivers).^2^ Older people and those from black and minority ethnic (BME) populations are at increased risk of stroke,^3^ ^4^ and as numbers of older people from diverse communities grow, it is becoming increasingly important to support the well-being of older, minority ethnic unpaid informal carers.^5^

Systematic reviews of research investigating informal caring post-stroke describe many challenges^6–8^ and support needs shared by carers across ethnic groups and differing care systems.^9^ Carers often report insufficient information and training about stroke and stroke services, particularly post-discharge from acute settings.^10^ Reviews highlight reduced quality of life, hypervigilance, anxiety, depression and having to cope with role and identity changes.^11–13^ A third of spousal carers report exhaustion and depression.^14^

However, there is considerable evidence that, in general, carers’ uptake of support services is low,^15–17^ even though using such services can make significant differences in delaying transfer to long-term care^18^ and improving carers’ quality of life.^19^

In developed countries, numbers of carers from BME groups in particular are rising as the demographic profiles of these populations age.^20^ In the UK, carers from BME groups provide proportionally more care than white British carers, and are also more likely to suffer from ill health.^21^ Despite this, evidence suggests that compared to their white counterparts, BME carers are less likely to use formal services,^22^ even though they express greater need for support.^23^ ^24^

Reasons for low service access are complex and multifaceted. For example, health and social care service uptake is influenced by cultural beliefs in terms of, for example, stigma associated with some health conditions.^23^ A recent systematic review identified several main explanations for carers’ failure to access and engage with services which included a mixture of attitudinal (eg, a sense of duty) and practical explanations. Importantly, while such reasons may apply to all ethnic groups, their impact may be exacerbated by language difficulties or information provided in culturally inappropriate ways.^25^ However, conclusions from research are limited by frequently failing to include majority groups for comparison, making it difficult to know if reasons for low uptake are restricted to minority ethnic groups or if they apply more widely.

The project, therefore, explored BME and white British carers’ experiences of accessing and receiving social care services in the community, focusing on similarities and differences between ethnic groups.

## Methods

The findings reported here are part of a larger qualitative project investigating carers’ satisfaction with social care. Findings relating to the impact of the heterogeneity of the focus group participants on group interaction are reported elsewhere where the focus was on, for example, identifying consensus or disagreement among the participants, rather than focusing on the content as presented here.^26^ The research approach draws on ethnography with an emphasis on the meanings participants give to actions and behaviour,^27^ and attempts to ensure that what participants said was not taken out of context from what they had said previously, or from what was said by others in the group.

Participants were recruited from voluntary organisations focusing on carers, BME groups or stroke survivors in and around London, UK. Some recruitment was face-to-face with the researchers visiting the organisations during, for example, carer support groups. In other cases, the organisations distributed the study information, potential participants responded to them and the organisations also set up the focus groups. Convenience sampling was used because recruitment proved difficult because of carers' caring responsibilities. To take part, participants had to be either currently or to have recently been caring for stroke survivors living in the community. This could include family members or friends. They also needed to be aged over 45 years old. This age group was selected to reduce some of the variability in the participants making comparison between the ethnic groups easier. They also had to self-identify as black African, black Caribbean, Asian Indian, Asian Pakistani or white British and to have used social care. In this context, social care refers to support offered in the community by statutory, commercial and voluntary sectors, and includes personal care, day centres, respite and support groups. It includes services aimed at both supporting carers and the person they care for. It does not include services provided in long-term care facilities and care homes.

Focus groups were conducted in English, audio-recorded and facilitated by the research team using a topic guide. This guide was developed to improve understanding of carers’ experiences of accessing and receiving services either for themselves or the person they cared for. The topics explored were broad and intended to help the participants describe and discuss their experiences in as little or as much detail as they wanted. Topics included participants’ reflections of how services may have changed over time as well as how services could be improved. Written informed consent was obtained from all participants.

Audio-recordings were transcribed verbatim and anonymised with pseudonyms by the research team, and were checked for accuracy by others in the research team. Analysis and data collection were simultaneous, allowing refinement of the topic guide and later data collection.^28^ Data were entered into NVivo V.10 and analysed thematically,^29^ permitting identification of both commonalities and differences between ethnic groups.

Using open coding, three members of the research team independently coded a selection of transcripts, reading and re-reading them to ensure familiarity with the data. Open codes were then discussed by the research team and a preliminary coding framework developed. This was repeated with further transcripts and the categories identified were refined and reduced in number by grouping them together. Following development of the final framework, the remaining transcripts were coded and the original ones recoded. All transcripts were analysed independently at least twice by two different researchers.

## Results

The seven focus groups averaged 80 minutes (50–90) and included between three and eight participants. In total, there were 41 carers aged 45–74 years. The majority (80%) were over 50 years. Just over two-thirds were women (71%) and a similar proportion were spouses (68%). Others were primarily adult children or daughters-in-law. They had been carers for between 14 months and 20 years.

Two focus groups included only South Asian carers with seven participants in each, two only white British carers (average four participants), and one only black African and black Caribbean participants (seven participants). The remaining two focus groups included participants from several ethnic groups (average six participants) (table 1).

**Table 1:** Participant demographics

		Age category		Gender		Relationship	
Focus group (n)	Ethnic group (n)	<50 years	50+ years	Female	Male	Spouse	Other
1 (7)	Asian Indian (5)						
	Asian Pakistani (2)	2	5	5	2	6	1
2 (7)	Asian Indian (7)	1	6	4	3	2	5
3 (5)	White British (5)	0	5	2	3	4	1
4 (3)	White British (3)	0	3	3	0	3	0
5 (7)	Black African (4)						
	Black Caribbean (3)	3	4	5	2	2	5
6 (5)	Asian Pakistani (2)						
	Asian Indian (1)						
	Black Caribbean (1)	0	5	3	2	4	1
	White British (1)						
7 (7)	Asian Indian (4)						
	Black African (1)						
	Black Caribbean (1)	2	5	7	0	7	0
	White British (1)						
Total (41)		8	33	29	12	28	13

Several interconnected themes were identified. Some themes related specifically to ethnicity, culture and language but others highlighted common experiences across ethnic groups. These themes are described below with anonymised quotes.

### Gap between hospital discharge and home

Irrespective of ethnic group, for most carers, the period after discharge following stroke was very difficult and was exacerbated by poor communication between services. Many carers were still suffering from the initial shock of the stroke and felt abandoned and ill-prepared. The apparent dearth of both health and social care services after discharge left carers feeling lost and overwhelmed, and the stark contrast between the support in hospital and limited community services heightened their sense of abandonment.

That panic sets in when you leave. It's like a bereavement leaving hospital. (Graham, white British male)

When people are discharged there is definitely a gap. I mean I don't have an English or language problem but there is a big gap in the services once you've been discharged from hospital. (Samiya, Asian Indian female)

Participants frequently commented on poor communication both within and between services which hindered accessing support and widened the perceived service gap.

In this day and age, there's got to be notes passed on. Where is the computer?…Why is it that you speak to one social worker, it could be ‘Oh she's on a course’ or ‘Oh she's away for a couple of days’. Before you know it, two or three days becomes two or three weeks, then it becomes a month. (Omar, Asian Indian male)

Carers often talked interchangeably about health and social care services, sometimes struggling to distinguish between them. Overall, they saw the ‘system’ as confusing and opaque. Unfamiliar terminology made services difficult to navigate.

When you've come out of hospital, at first there's absolutely nothing…Nobody tells you anything, or if they do, they are using terminology that you've never experienced before…I didn't feel there was anybody there that spoke my language. (John, white British male)

### Carers as persistent advocates—knowing the system and fighting for support

In order to access services, carers across ethnic groups felt they had to fight ‘the system’. They repeatedly commented on the necessity for ‘speaking loudly’, being tenacious and ‘knowing the system’.

If you don't make a song and a dance about…you need to put yourself forward. And there are people who doesn't know, and they are not talking, so they are not getting any help….I sort of really fought for it…then the ball started rolling…I got my ramp… (Upma, Asian Indian female)

Carers often had multiple responsibilities and wanted increased support but felt their requests were ignored, either because services did not fully understand their difficult circumstances, or because they were deemed low priority.

Mother had a hip replacement on a Wednesday, Dad had his by-pass on the Friday. I couldn't have anyone to look after her because all our family live far. So then I was constantly on the phone trying to get some help but they just don't listen, they don't care…well it's a priority thing, so obviously there's people worse off than you. (Khayrah, Asian Indian female)

### Balancing the effort in accessing services with their needs and poor or unsuitable services

Carers from all the included ethnic groups reported that accessing and engaging with services required huge effort. Their difficulties were exacerbated by unresponsive services and large amounts of paperwork. Communicating with service providers was very challenging leading to anger, stress and frustration, furthering carers’ already hard circumstances. Paperwork was described as lengthy and ‘horrendous’ adding to the considerable time required to access services. Assessments often came to nothing, adding to their frustration.

The thing is, you pour your emotions out, but they just put it down on a paper. It's not the same…what I say & the way they write it down is two different things. (Sathinder, Asian Indian male)

Stroke survivors’ needs, choices and abilities played an important part in whether carers accessed services. Sometimes this related to stroke survivors’ pre-stroke personality or age but often the stroke had affected what they benefitted from or enjoyed. For example, although support groups were generally viewed positively, carers sometimes highlighted post-stroke changes which made tolerating large numbers of people difficult.

[After the stroke] my husband didn't like talking all the time or noise or anything. (Raameen, Asian Pakistani female)

Once accessed, services were frequently seen as poor, adding to carers’ ambivalence about using them.

One girl came in and she took one rag. Mum had two face flannels. And I was looking at them, not saying anything. She washed [mimes washing her face]. The next thing you know, she went with the same rag, down [mimes washing bottom half of body]. (Evelyn, black Caribbean female)

This ambivalence meant they often gave up trying to access them.

You get tired. Because many of the times, you call, and they don't give you the answer. They don't meet their promise…sometimes you reach a point where you get tired of it. (Marina, black African female)

Persistence or abandoning efforts to access and engage with services was also influenced by carers’ belief that they were the best person to provide care.

### Carers as the best person to care

Carers from all ethnic groups, but particularly from BME groups, emphasised not only their desire to look after the stroke survivor but also the perception that they were usually the best person to care. This was explained by their unique, long-term relationship with stroke survivors and the belief that they, unlike paid care workers, genuinely care. This meant they were often ambivalent about asking for support.

But what you have to do for your wife and parents, no one can care…I have a son… he suffers from mental illness as well so I'm looking after two disabled people. But I'm happy. No complaints. I'm very, very happy. I'm doing something for my family. As Peter (another participant) said ‘What you can do for your relatives, no one else can do. They [care workers] have no feeling. (Abdul, Asian Indian male)

Another concern about paid care workers was their apparent constant rushing and the short time allocated. As a result, the care tasks were often perceived not to be done properly. Short visits also left limited time to develop relationships and interact with stroke survivors which was particularly important if care workers were providing personal care. By contrast, family members could take as long as needed.

They are rushing. And they need to take time…and I say, ‘Hold on. Patient with my mum, she can't turn like that, take time.’ (Evelyn, black Caribbean female)

Some BME carers saw their desire to care as culturally influenced.

We're Muslims, so we do look after our elderly anyway at the end of the day. It's only in extreme circumstances that they'll be put in to care homes. (Omar, Asian Indian male)

This was also sometimes specifically related to cultural perceptions of family duty. During a discussion about their Asian culture, one wife said:

You worry for the partner. If one is weak, you have to look after the other one. To do with duty and love. (Raameen, Asian Pakistani female)

However, other carers highlighted family relationships, rather than cultural expectations. This participant emphasised she was doing what most wives would do.

I mean we've never asked for anything, we've been married for 60 years and I just look after him the best that I can. (Freda, white British female)

Similarly, this man emphasised their relationship.

In the end she's my wife….it's my responsibility to look after her. (Abdul, Asian Indian male)

### Cultural aspects of caring: ethnicity, culture, religion and language

Some discussions about social care were clearly and specifically related to ethnicity and culture. The commonest of these concerned communication and accessing support in a second language. This was usually, but not exclusively, discussed by South Asian carers and included not only language differences but also other facets of communication.

We are Guajarati and somebody coming in—English people—they don't understand same culture, same things. (Chetna, Asian Pakistani female)

Sometimes cultural differences related to the manner with which different groups communicated, for example, being uncomfortable talking about intimate health issues such as continence.

I didn't know we could get pads on the NHS. For months and months we kept quiet and didn't tell anybody and we coped ourselves and it was very, very hard for my Dad ….We're very reserved people, Asian people, you know. And we don't know how to communicate, how to share information. (Samiya, Asian Indian female)

Differences in non-verbal styles of expression were mentioned by some black participants for sometimes hampering rapport and interpersonal communication. Talking more loudly than other ethnic groups was highlighted as potentially appearing rude.

So there are differences between the black and the white culture…Because what I feel is respect, might not be respect for you. Or the way I would talk to you, another person might think ‘Oh she is not respectful, she is not respecting me. (Nancy, black Caribbean female)

Among the Asian participants, having cultural backgrounds and languages in common with care workers was perceived as facilitating communication and understanding. This made it easier for carers to express their needs, but it also influenced their trust in services.

There are more and more agencies coming in now and they know the need of the Asians, and the other peoples. So they cater for the people… for instance my wife wants someone Urdu speaking, or as the sister said [indicating another participant] Guajarati speaking. (Abdul, Asian Indian male)

Additionally, care workers from their own culture were sometimes perceived as being more like family members providing better, more caring support.

We pay you money only but money is not only important. They give you heart as well. Love. Just like daughter and Mum. (Chetna, Asian Pakistani female)

Muslim carers emphasised that non-Muslim care workers often appeared not to understand the importance of their religious observances, highlighting praying and washing rituals. Employing Muslim care workers made explanations unnecessary.

So they [non-Muslim care workers] don't know about our needs, our religious needs and stuff like that. So obviously, like when she's trying to do her ablution she constantly has to explain to them, what she needs to do and like they can't understand, they think it's a bit stupid or a bit petty. (Khayrah, Asian Indian female)

On the other hand, care workers from their own ethnic background did not guarantee good support.

This lady, well she was Asian, from my own country. She was supposed to do the mopping in the kitchen…What she would do, she would just soak the mop in the kitchen sink instead of using the bucket. (Abdul, Asian Indian male)

It was also suggested that care workers sometimes took advantage of families from their own background, knowing they were less likely to complain if, for example, they left early.

Afterwards I knew from other carers, he was doing it every job, you know. And because he was a Bengali, and he was working for other Bengali people…and they used to put up with it because he was the same… (Usha, Asian female)

In some cases, carers described how short, rushed visits interfered with religious observances.

They might have a 20 minute time slot to prepare her lunch. But if she's got to pray and she's got to go and do her Wudu [washing ritual] and she needs a bit of help…they are pushed for time. (Omar, Asian Indian male)

However, other carers emphasised issues common to all service users and the importance of treating everyone as individuals.

How would we like to be treated? That's got to be the philosophy…‘What would I want?’ That should be the focus of it. (Peter, black Caribbean male)

## Discussion

Carers from all included ethnic groups highlighted the challenges they face, while simultaneously emphasising their frequent ambivalence about accessing and engaging with services. The belief that families are best placed to provide support for the stroke survivors and that services are frequently unsuitable, often resulted in failure to access or engage with services. Opaque systems, unfamiliar terminology and onerous paperwork meant they were quick to abandon efforts to access support.

This struggle to access services can add to the ‘burden’ of their role,^30^ and may help explain carers’ reported low service uptake.^15–17^ However, the tendency to perceive this as a problem situated within carers rather than in service provision, needs to change. More attention should be directed at unresponsive services failing to appreciate carers’ changing needs and carers’ huge efforts required to access them. The negative impact on carers and their consequent exhaustion and helplessness needs to be better recognised. The period immediately following discharge from hospital is often when carers need the most support, and yet this is when services are found most wanting. This service gap has been identified before^31^ but our research highlighted poor communication across and within health and social care adding to the perceived gap. Improved multidisciplinary working, with all disciplines working closely with carers prior to discharge is essential if the gap is to be bridged.

There were many similarities in experiences across ethnic groups here, but the South Asian carers stood out from other carers in several ways. First, supporting earlier research,^32^ the additional challenges of accessing services in a second language were highlighted. Second, again supporting other research,^33^ ^34^ some Asian carers also said they would have preferred support staff from their own cultural or religious backgrounds. This appeared to be particularly important when it came to personal care and understanding, respecting and supporting their religious observances. It was also expected to help provide insight into cultural preferences in, for example, food preparation. However, matching care worker religion or ethnicity may not always be practical, but it may be worth considering it particularly in relation to personal care and religious observances. However, this sort of matching may set up false expectations. Indeed, several participants expressed disappointment when such care workers had failed to live up to expectations.

As described elsewhere,^35^ explanations for why carers had taken on their caring role differed across ethnic groups. South Asian participants tended to highlight their culture or religion. These participants emphasised family ‘duty’ as part of their culture or religion, while participants from other ethnic groups were more likely to suggest that this is simply what family members do for each other, rather than having an explicit cultural basis. This is important because taking on a caring role out of a sense of duty rather than choice and ‘love’ affect how the role is perceived, in turn influencing support needs.^36^ Furthermore, this may have an impact on both initial attempts to access services and how persistent carers are in remaining engaged with services. Additionally, it may influence how long carers feel able to continue caring at home. In relation to the literature, it may also demonstrate how perceptions of cultural differences in the reasons for taking on the caring role and, as a consequence, accepting and engaging with services, might be exaggerated. Even those carers here (mostly white British) who did not explicitly state that being a carer was their cultural duty or responsibility, were responding to their cultural norms. Families worldwide often take on caring roles and are usually unwilling to place family members in institutional care citing many reasons, not all explicitly culturally related. Rather than emphasising ethnic differences, it is important to value what carers do, while recognising that many want the caring role and find it rewarding.^37^

### Strengths and limitations

Strengths of this study include the inclusion of white ‘majority’ participants which permitted direct comparison with minority ethnic group participants. The in-depth nature of the focus group discussions with over 40 participants also added to our understanding of their experiences with services. However, all focus groups were conducted in English excluding carers with limited or no English. This is important as these carers are likely to find accessing services more challenging than those with a good command of English. Participants were all recruited from the voluntary sector and had, therefore, engaged with services. Ideally, future research would include carers who have not accessed any support services as this would add an important perspective. Finally, we only investigated five ethnic groups, which are clearly very broad categories with inherent limitations.

### Conclusions

In conclusion, older carers find interfacing with services challenging and need persistence and expertise to navigate the system and access support. Carers from all groups want to be treated as individuals whether this relates to their culture or other personal characteristics. The processes required to access services should be improved for carers from all ethnic groups, but the extra challenges for carers from BME groups need recognition. Perhaps specific points in the caring trajectory, such as post-stroke reviews like those undertaken in the UK at regular intervals after discharge,^38^ could be identified to ensure that carers are proactively asked about their support needs. Generally, carers want their role and feel they are best placed to provide the individualised support their loved-ones need. Services, therefore, need to demonstrate how they can work with families to support them.

## References

[1] Mackay J, Mensah G The atlas of heart disease and stroke. Geneva: World Health Organisation, 2004 http://www.who.int/cardiovascular_diseases/resources/atlas/en/ (accessed Jul 2015).

[2] National Audit Office. Progress in improving stroke care. London: Stationery Office, 2010.

[3] Stroke Association. Stroke statistics 2013. https://www.stroke.org.uk/sites/default/files/stroke_statistics_2015.pdf (accessed 13 Oct 2015).

[4] Markus HS, Khan U, Birns J Differences in stroke subtypes between black and white patients with stroke: the South London Ethnicity and Stroke Study. Circulation 2007;116:2157–64. 10.1161/CIRCULATIONAHA.107.699785 17967776

[5] Department of Health: Caring for our future. Reforming care and support. London: HM Government, 2012.

[6] Greenwood N, Mackenzie A, Cloud GC Informal carers of stroke survivors—factors influencing carers: a systematic review of quantitative studies. Disabil Rehabil 2008;30:1329–49. 10.1080/09638280701602178 19230230

[7] Greenwood N, Mackenzie A, Cloud GC Informal primary carers of stroke survivors living at home—challenges, satisfactions and coping: a systematic review of qualitative studies. Disabil Rehabil 2009;31:337–51. 10.1080/09638280802051721 18608402

[8] Quinn K, Murray C, Malone C Spousal experiences of coping with and adapting to caregiving for a partner who has a stroke: a meta-synthesis of qualitative research. Disabil Rehabil 2014;36:185–98. 10.3109/09638288.2013.783630 23597001

[9] Perry L, Middleton S An investigation of family carers’ needs following stroke survivors’ discharge from acute hospital care in Australia. Disabil Rehabil 2011;33:1890–900. 10.3109/09638288.2011.553702 21314290

[10] Mackenzie A, Perry L, Lockhart E Family carers of stroke survivors: needs, knowledge, satisfaction and competence in caring. Disabil Rehabil 2007;29:111–21. 10.1080/09638280600731599 17364762

[11] McGurk R, Kneebone I The problems faced by informal carers to people with aphasia after stroke: a literature review. Aphasiology 2013;27:765–83. 10.1080/02687038.2013.772292

[12] Greenwood N, Mackenzie A Informal caring for stroke survivors: meta-ethnographic review of qualitative literature. Maturitas 2010;66:268–76. 10.1016/j.maturitas.2010.03.017 20430542

[13] Green TL, King KM Experiences of male patients and wife-caregivers in the first year post-discharge following minor stroke: a descriptive qualitative study. Int J Nurs Stud 2009;46:1194–200. 10.1016/j.ijnurstu.2009.02.008 19303597

[14] Berg A, Palomaki H, Lonnquist J Depression among caregivers of stroke survivors. Stroke 2005;36:639–43. 10.1161/01.STR.0000155690.04697.c0 15677575

[15] Toseland RW, McCallion P, Gerber T Predictors of health and human services use by persons with dementia and their family caregivers. Soc Sci Med 2002;55:1255–66. 10.1016/S0277-9536(01)00240-4 12365535

[16] Brodaty H, Thomson C, Thompson C Why caregivers of people with dementia and memory loss don't use services. Int J Geriatr Psych 2005;20:537–46. 10.1002/gps.1322 15920707

[17] Stockwell-Smith G, Kellett U, Moyle W Why carers of frail older people are not using available respite services: an Australian study. J Clin Nurs 2010;19:2057–64. 10.1111/j.1365-2702.2009.03139.x 20920032

[18] Gaugler JE, Kane RL, Kane RA Community based service utilization and its effects on dementia caregiving. Gerontologist 2005;45:177–85. 10.1093/geront/45.2.177 15799982

[19] Winslow BW Effects of formal supports on stress outcomes in family caregivers of Alzheimer's patients. Res Nurs Health 1997;20:27–37. 10.1002/(SICI)1098-240X(199702)20:1<27::AID-NUR4>3.0.CO;2-W 9024475

[20] Lievesley N The future ageing of the ethnic minority population of England and Wales. London: Centre for Policy on Ageing and the Runnymede Trust, 2010.

[21] Carers UK. Half a million voices: improving support for BAME carers. London: Carers UK, 2011.

[22] Dunlop D, Manheim L, Song J Gender and ethnic/racial disparities in health care utilization among older adults. J Gerontol B Soc Sci 2002;57:S221–33. 10.1093/geronb/57.4.S221 12084792

[23] Giunta N, Chow J, Scharlach AE Racial and ethnic differences in family caregiving in California. J Human Beh Soc Environ 2004;9:85–109. 10.1300/J137v09n04_05

[24] Scharlach AE, Kellman R, Ong N Cultural attitudes and caregiver service use: lessons from focus groups with racially and ethnically diverse family caregivers. J Gerontol Soc Work 2006;47:133–56. 10.1300/J083v47n01_09 16901881

[25] Greenwood N, Habibi R, Smith R Barriers to access and minority ethnic carers’ satisfaction with social care services in the community: a systematic review of qualitative and quantitative literature. Health Soc Care Community 2015;23:64–78. 10.1111/hsc.12116 25135207 PMC4283974

[26] Greenwood N, Ellmers T, Holley J An exploration of the influence of ethnic group composition on focus group discussions. BMC Res Methodol 2014;14:107 10.1186/1471-2288-14-107 PMC4177596 25240807

[27] Hammersley M, Atkinson P In: Ethnography: Principles in practice. 2nd edn London: Routledge, 1995.

[28] Pope C, Ziebland S, Mays N Analysing qualitative data. In: Pope C, Mays N, eds. Qualitative research in health care. 2nd edn London: BMJ Books, 1999:75–88. 10.1136/bmj.320.7227.114 PMC1117368 10625273

[29] Braun V, Clarke V Using thematic analysis in psychology. Qual Res Psychol 2006;3:77–101. 10.1191/1478088706qp063oa

[30] McPherson KM, Kayes NK, Moloczij N Improving the interface between informal carers and formal health and social services: a qualitative study. Int J Nurs Stud 2014;51:418–29. 10.1016/j.ijnurstu.2013.07.006 23928324

[31] Tyson S, Turner G Discharge and follow-up for people with stroke: what happens and why. Clin Rehab 2000;4:381–92. 10.1191/0269215500cr331oa 10945422

[32] Merrell J, Kinsella F, Murphy F Support needs of carers of dependent adults from a Bangladeshi community. J Adv Nurs 2005;51:549–57. 10.1111/j.1365-2648.2005.03539.x 16129005

[33] Fazil Q, Bywaters P, Ali Z Disadvantage and discrimination compounded: the experience of Pakistani and Bangladeshi parents of disabled children in the UK. Disabil Society 2002;3:237–53. 10.1080/09687590220139838

[34] Hensel E, Krishnan M, Saunders K Impact of policy shifts on South Asian carers in the United Kingdom. J Policy Pract Intellect Disabil 2005;2:10–17. 10.1111/j.1741-1130.2005.00003.x

[35] Williams C, Johnson MRD Race and ethnicity in a welfare society. Maidenhead, UK: Open University Press, 2010.

[36] Lawrence V, Murray J, Kritika Samsi K Attitudes and support needs of black Caribbean, south Asian and white British carers of people with dementia in the UK. B J Psychiat 2008;193:240–6. 10.1192/bjp.bp.107.045187 18757985

[37] Mackenzie A, Greenwood N Positive experiences of caregiving in stroke: a systematic review. Disabil Rehab 2012;34:1413–22. 10.3109/09638288.2011.650307 22309576

[38] Department of Health. National stroke strategy. London: DH, 2007 http://www.dh.gov.uk/en/Publicationsandstatistics/Publications/PublicationsPolicyAndGuidance/DH_081062 (accessed 13 Oct 2015).

